# *Leptolegnia chapmanii* como alternativa biológica para el control de *Aedes aegypti*

**DOI:** 10.7705/biomedica.4598

**Published:** 2019-12-30

**Authors:** Manuel E. Rueda, Isabella Tavares, Claudia C. López, Juan García

**Affiliations:** 1 Laboratorio de Hongos Entomopatógenos y Control Biológico, Centro de Estudios Parasitológicos y de Vectores (CEPAVE), Universidad Nacional de La Plata-CONICET, Buenos Aires, Argentina Universidad Nacional de La Plata Laboratorio de Hongos Entomopatógenos y Control Biológico Centro de Estudios Parasitológicos y de Vectores Universidad Nacional de La Plata Buenos Aires Argentina; 2 Consejo Nacional de Investigaciones Científicas y Técnicas (CONICET), Buenos Aires, Argentina Consejo Nacional de Investigaciones Científicas y Técnicas Buenos Aires Argentina; 3 Comissão Técnica de Combate ao Aedes spp. no Campus “Luiz de Queiroz”, Laboratório de Controle Microbiano de Insetos, Departamento de Entomología, Escola Superior de Agricultura Luiz de Queiroz, Piracicaba, Brasil Laboratório de Controle Microbiano de Insetos Departamento de Entomología Superior de Agricultura Luiz de Queiroz Piracicaba Brasil; 4 Comisión de Investigaciones Científicas, Buenos Aires, Argentina Comisión de Investigaciones Científicas Buenos Aires Argentina

**Keywords:** Aedes, control biológico de vectores, mosquitos vectores, vectores de enfermedades, salud pública, Aedes, pest control, biologica, mosquito vectors, diseases vectors, public health

## Abstract

*Leptolegnia chapmanii* es un microorganismo patógeno facultativo de diversas especies de mosquito, entre las que se destacan, por su importancia médica y sanitaria, especies de los géneros *Aedes*, *Culex* y *Anopheles*. El potencial de *L. chapmanii* como alternativa de control radica en la virulencia, capacidad patógena y grado de especificidad que presenta hacia los estadios larvales de las diferentes especies de mosquito, y por su inocuidad frente a organismos acuáticos no blanco como, por ejemplo, peces y anfibios. Su presencia natural ha sido reportada en Argentina, Brasil, y Estados Unidos, pensándose como posible en otros países dentro del continente americano. La eficacia de *L. chapmanii* como controlador se ve influenciada por factores externos, como la temperatura, la radiación y el pH, entre otros.

Uno de los objetivos de trabajo del Grupo de Hongos Entomopatógenos del Centro de Estudios Parasitológicos y de Vectores de la Universidad Nacional de La Plata, corresponde al desarrollo de protocolos para la producción, formulación, almacenamiento y aplicación de productos basados en este microorganismo. Con este referente, estamos desarrollando un proyecto con *L. chapmanii* que se encuentra en la fase inicial, en la que se está trabajando la prueba de concepto a escala de laboratorio. Se espera continuar en el futuro con estudios de eficacia, eficiencia, estabilidad y seguridad ecotoxicológica, a diferentes escalas.

## Abstract (en)

*Leptolegnia chapmanii* is a facultative pathogen of many species of mosquitoes, among which species of the genus *Aedes, Culex* and *Anopheles* stand out for their medical and sanitary importance. The potential of *L. chapmanii* as an alternative to control lies in its virulence, pathogenicity and specificity against the larval stages of mosquitoes, and because of its harmlessness to non-target species such as fish and amphibians, among others. The natural presence of *L. chapmanii* had been reported in Argentina, Brazil and the United States. Its presence is possible in other countries throughout the American continent.

The development of protocols to produce, formulate, store and apply products based on this microorganism is one of the objectives proposed for the group of Entomopathogenic Fungi at the *Centro de Estudios Parasitológicos y de Vectores, Universidad Nacional de La Plata*. The efficacy of *L. chapmanii* as controller is affected by external factors such as temperature, pH, salinity and radiation among others. The process of transfer from the research centers to industry implies many phases. In this way, our project with *L. chapmanii* is in an initial phase, where we are working on a laboratory scale in proof of concept. We hope to begin soon with the efficacy, efficiency, stability and ecotoxicological safety tests, at the laboratory, semi-field and field scale.

En este documento se recopila la información referente a *Leptolegnia chapmanii* (Straminipila: Saprolegniales), se presenta su historia como agente entomopatógeno y se resalta su potencial como alternativa viable y complementaria para el control de *Aedes aegypti*, entre otras especies de mosquitos*.*

Su intención es dar a conocer a este microorganismo e incentivar su prospección en ambientes naturales, con el fin de ampliar el conocimiento de su distribución geográfica. Además, se presenta el enfoque de trabajo por parte de nuestro grupo de investigación, en relación con el aislamiento argentino de *L. chapmanii*, así como algunas consideraciones desde nuestra experiencia y perspectiva.

## Clasificación taxonómica

*Leptolegnia chapmanii* (Seymour) está entre los microorganismos conocidos comúnmente como hongos acuáticos o pseudohongos, denominados también oomicetos (‘hongo huevo’) por desarrollar estructuras reproductivas redondas en forma de huevo.

En general, los oomicetos han evolucionado de forma tal que en la actualidad se encuentran ampliamente distribuidos, habitando diversos ambientes terrestres y acuáticos ^1^. Estos microorganismos fueron clasificados durante mucho tiempo dentro del reino de los hongos, por la similitud en las estructuras de hifas que desarrollan y por obtener su alimento mediante el proceso de absorción ^2^.

La microscopía electrónica y las técnicas moleculares desarrolladas durante el siglo XX, permitieron reclasificar los oomicetos al demostrarse diferencias con los ‘hongos verdaderos’ en aspectos como:

- poseer celulosa como principal componente de la pared celular (quitina en el caso de los hongos);
- presentar un núcleo diploide en su tejido vegetativo (haploide o dicariótico en los hongos);
- poseer mitocondrias con crestas tubulares y no planas como en los hongos, y
- desarrollar zoosporas con dos flagelos heterocontos (desiguales entre sí), cuando los hongos que los producen solo poseen uno ^(3)^.

Es así que, en la actualidad, los oomicetos (Peronosporomicetos) se clasifican en el reino Straminipila (Chromista) ^4^^-^^7^, e incluyen especies con hábitos saprófitos, así como patógenos que afectan plantas (por ejemplo, *Phytophthora* spp.), animales (por ejemplo, *Saprolegnia* spp. y *Aphanomyces* spp.) o ambos ^8^^-^^12^.

La clasificación taxonómica de los Peronosporomicetos ha sido revisada y organizada varias veces, y la relación filogenética propuesta entre las especies integrantes es tema de controversia ^7^^,^^13^^-^^16^. Tradicionalmente, se han reconocido cuatro órdenes dentro de los Peronosporomicetos (Lagenidiales, Leptomitales, Peronosporales y Saprolegniales) con 50 géneros, aproximadamente ^17^. Se conocen cerca de 676 especies de los Peronosporomicetos, y se estima que pueden existir entre 1.000 y 10.000 especies ^18^. Además, *L. chapmanii* se encuentra subclasificada en lo que se conoce como “galaxia de los Saprolegniales” ^1^.

## Morfología y ciclo de vida

*Leptolegnia chapmanii* ha sido encontrado en ambientes dulceacuícolas y es un agente patógeno facultativo de los estados acuáticos de diversas especies de mosquitos.

**Figura 1.**
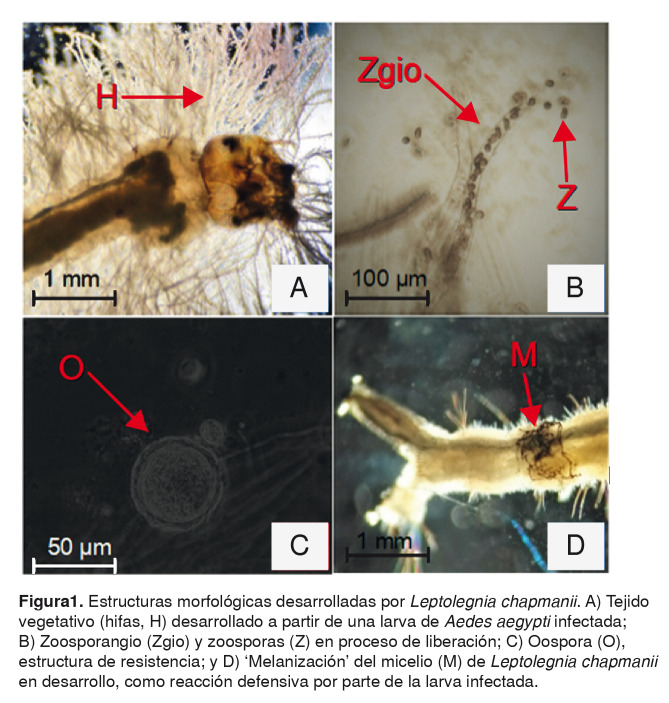
Estructuras morfológicas desarrolladas por *Leptolegnia chapmanii*. A) Tejido vegetativo (hifas, H) desarrollado a partir de una larva de *Aedes aegypti* infectada; B) Zoosporangio (Zgio) y zoosporas (Z) en proceso de liberación; C) Oospora (O), estructura de resistencia; y D) ‘Melanización’ del micelio (M) de *Leptolegnia chapmanii* en desarrollo, como reacción defensiva por parte de la larva infectada.

Su desarrollo, tanto en medio de cultivo como a partir de las larvas de mosquito, presenta un crecimiento micelial, con desarrollo de hifas no segmentadas (figura 1A). Al agotarse su fuente alimenticia, desarrollan estructuras reproductivas móviles denominadas ‘zoosporas’ (figura 1B), las cuales nadan activamente durante varios minutos, dispersándose en el ambiente en busca de nuevos sustratos, antes de enquistarse y quedar a la deriva. Estas estructuras no suelen vivir por periodos prolongados; como referencia se sabe que, en condiciones de laboratorio, han permanecido viables hasta por 50 días ^19^.

Cuando las condiciones ambientales son hostiles, *L. chapmanii* desarrolla por meiosis gametos femeninos y masculinos (oogonios y anteridios, respectivamente) los que, al fusionarse, dan origen a estructuras de resistencia conocidas como ‘oosporas’ ^20^ (figura 1C). Estas estructuras pueden persistir en el ambiente hasta que las condiciones ambientales de temperatura y humedad, entre otras, sean adecuadas y, entonces, se activan metabólicamente para producir zoosporas que permiten su dispersión ^21^.

Su ciclo de vida como agente patógeno se inicia cuando las zoosporas son ingeridas por las larvas del mosquito o cuando entran en contacto tópicamente con la cutícula larvaria. Posteriormente, el microorganismo penetra hasta la cavidad celómica, en donde se desarrolla a expensas de los tejidos. La ‘melanización’ de las hifas en desarrollo (figura 1D) sirve como signo de la infección y es consecuencia de la reacción de defensa por parte de las larvas frente al patógeno ^22^^-^^24^. Dependiendo de lo masiva que sea la infección, las larvas mueren entre 8 y 72 horas después de entrar en contacto con las zoosporas ^25^. Una vez que el agente patógeno ha invadido todo el cuerpo de las larvas y se terminan los nutrientes, el micelio se desarrolla externamente y se inicia un proceso de división celular (asexual) que da origen a los zoosporangios (figura 1B); en estos, se forman las zoosporas y desde ellos se liberan para dar inicio nuevamente al ciclo.

## Distribución geográfica actual y especies de mosquitos asociadas

Este microorganismo fue reportado por primera vez en Luisiana, Estados Unidos, durante los años 70 del siglo XX; se encontró en larvas del mosquito *Aedes triseriatus* (Say) presentes en el agua almacenada en el hueco de un árbol ^26^. Posteriormente en la misma década, se encontró en el estado de Carolina del Sur (Estados Unidos) parasitando larvas de *Culex pipiens quinquefasciatus* (Say) desarrolladas en charcos en suelo de tierra, dentro de las instalaciones de un criadero experimental de mosquitos ^27^. Existen dos aislamientos norteamericanos adicionales que datan de los años 80 (siglo XX) y que fueron realizados a partir de larvas de *Mansonia titillans* (Wa0lkers) presentes en pozos abandonados en dos localidades en Florida ^28^.

En 1996, se reportó al sur del continente americano, en cercanías a la ciudad de La Plata, provincia de Buenos Aires, Argentina, parasitando larvas de *Ochlerotatus albifasciatus* (Macquart) presentes en un cuerpo de agua temporal desarrollado en un área rural ^29^. El último reporte de ocurrencia natural corresponde al año 2014 y procede de la ciudad de Posadas en la provincia de Misiones en Argentina, en donde el personal de vigilancia y control de la municipalidad lo encontró en larvas de *A. aegypti* presentes en un balde con aguas lluvia en un sector urbano (Rueda ME, Montero G, Gauto N, Tejerina F, Micieli MV, García JJ, *et al*. Nuevos registros de aislamientos de *Leptolegnia chapmanii*, agente patógeno de larvas de mosquitos (Diptera: Culicidae) para la provincia de Misiones. IX Congreso Argentino de Entomología, Posadas, 2015).

El reporte más reciente de *L. chapmanii* (entre varios oomicetes entomopatógenos) corresponde al 2015, procedente de la región central de Brasil ^30^. En este caso, el hallazgo no correspondió a una ‘infección natural’ resultante de la interacción entre el agente patógeno y los estadios acuáticos de alguna especie de mosquito con presencia natural en esos ambientes.

**Figura 2.**
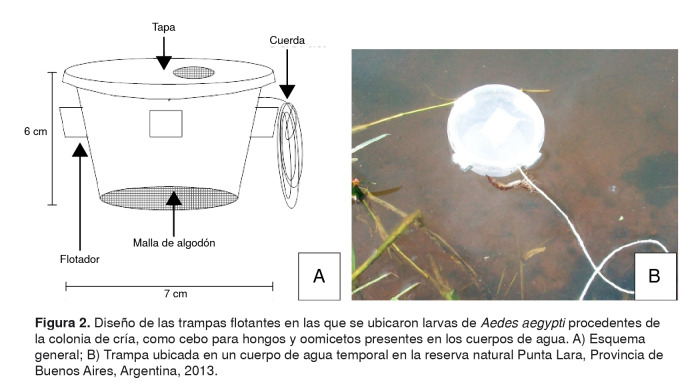
Diseño de las trampas flotantes en las que se ubicaron larvas de *Aedes aegypti* procedentes de la colonia de cría, como cebo para hongos y oomicetos presentes en los cuerpos de agua. A) Esquema general; B) Trampa ubicada en un cuerpo de agua temporal en la reserva natural Punta Lara, Provincia de Buenos Aires, Argentina, 2013.

Su hallazgo fue el resultado de un proyecto en el que se realizó la prospección de hongos y oomicetos en ambientes acuáticos, empleando unas trampas flotantes con larvas de *A. aegypti*, procedentes de una colonia de cría, en su interior y a modo de cebo. Las trampas se ubicaron en diferentes cuerpos de agua (figura 2) por un periodo de entre 24 y 48 horas, lo que permitió la interacción entre las larvas y la microbiota presente. Los hongos y oomicetos patógenos se aislaron en el laboratorio a partir de las larvas muertas o con signos de infección y, posteriormente, se confirmó su actividad patógena sobre poblaciones larvales (L2/L3) sanas de *A. aegypti* procedentes de la colonia de cría (Santos KR, Montalva C, Rueda ME, Filgueira MD, Fernandes EK, Humber RA, *et al*. Atividade de fungos isolados de dípteros coletados em Goiás e Tocantins em *Aedes aegypti* e *Musca domestica*. Rev Patol Trop. 2016;45(Supl.1):70. XIV Seminário de Patología Tropical e Saúde Pública, Universidade Federal de Goiás)*.*

## Potencial de *Leptolegnia chapmanii* como agente para el control biológico de *Aedes aegypti* entre otros mosquitos

*Leptolegnia chapmanii* ha sido objeto de estudio y análisis desde que fue aislado por primera vez hace más de 40 años. Inicialmente, se determinó su actividad patógena sobre diferentes especies de mosquitos (subfamilias Culicine y Anopheline); de manera general, se encontró una mayor vulnerabilidad por parte de los estadios larvales más jóvenes (L1 y L2), así como una mayor resistencia ante la infección por parte de los más avanzados (L3, L4 y pupa) ^25^.

La capacidad patógena de los aislamientos de *L. chapmanii* ha sido ampliamente evaluada. Se conoce en la actualidad un rango de especies de mosquitos huéspedes ^31^^-^^34^, entre los que destacan varias especies de los géneros *Aedes* (principales transmisores de los virus del dengue, fiebre amarilla, Zika y chikungunya), *Culex* (transmisores de otros virus y parásitos) y *Anopheles* (transmisores del protozoo *Plasmodium* spp., causante de la malaria). Igualmente, se ha reportado su capacidad patógena sobre especies de mosca negra (Diptera: Simuliidae) ^35^, con importancia sanitaria por transmitir al nematodo causante de la oncocercosis y por generar reacciones alérgicas agudas ^36^.

Por otro lado, la inocuidad de *L. chapmanii* sobre organismos no blanco ha sido corroborada con especies como *Daphnia* sp. (Crustacea: Cladocera), *Hyalella curvispina* (Crustacea: Amphipoda), *Mesocyclops annulatus* (Crustacea: Cyclopoida), *Strelkovimermis spiculatus* (Nematoda: Mermithidae), *Cnesterodon decenmaculatus* (Vertebrata: Pisces), *Bufo arenarum* (Vertebrata: Amphibia), *Peltoperla* sp. (Insecta: Plecoptera), *Dicranola* sp. y *Tipula* sp. (Insecta: Diptera), y otras pertenecientes a las familias Coenagrionidae (Insecta: Odonata), Psychodidae y Ceratopogonidae (Insecta: Diptera), e Hydrophyllidae (Insecta: Coleoptera), que habitan naturalmente los ambientes en donde se desarrollan las larvas de algunos mosquitos con interés de controlar ^31^^,^^33^.

Se estudió su compatibilidad con productos larvicidas como el temefos y los basados en *Bacillus thuringiensis* var. *Israelensis* (*Bti*) y se encontró, no solo que las zoosporas de *L. chapmanii* no se ven afectadas por estos productos en las concentraciones de uso recomendadas, sino que su aplicación en conjunto presenta un efecto sinérgico, aumentándose la mortalidad de las larvas en comparación con lo obtenido cuando se aplican los productos por separado ^37^.

## Visión de trabajo con el aislamiento argentino de *Leptolegnia chapmanii*

La situación actual en el contexto global respecto a los mosquitos y las enfermedades asociadas a los virus y parásitos transmitidos, además del creciente interés por alternativas de control biológico más amigables con el ambiente, nos han llevado como grupo de investigación a trazarnos objetivos puntuales en torno a *L. chapmanii.* De esta manera, nuestro trabajo actual está enfocado en:

- Evaluar diferentes tecnologías y sustratos nutricionales ecológicos y económicos para la producción de *L. chapmanii*.
- Desarrollar protocolos de producción a escala piloto que puedan llegar a ser transferidos a la industria.
- Evaluar alternativas de formulación, con el fin de generar presentaciones sólidas y líquidas de *L. chapmanii*, que puedan ser almacenables y de manipulación y aplicación fácil y práctica.
- Desarrollar protocolos de utilización, evaluando diferentes tecnologías acordes con las presentaciones generadas.

A la fecha, no existen reportes en cuanto al desarrollo de productos formulados basados en este microorganismo, ni de procesos de transferencia desde los centros de investigación hacia la industria. El proceso de desarrollo de una intervención o un producto para el control de vectores, contempla múltiples etapas ^38^.

Por nuestra parte, podría decirse que el grado de avance del proyecto con *L. chapmanii*, se encuentra en una etapa temprana (fases 1 y 2), en la que ya se ha transitado parte del proceso, con resultados favorables a escala de laboratorio e información de base sólida, pero aún correspondiente a una ‘prueba de concepto’. Estamos trabajando en el desarrollo de metodologías para la producción de biomasa a mayor escala, así como en la formulación del ingrediente activo.

Para el futuro, contemplamos el desarrollo de las pruebas de eficacia, eficiencia, estabilidad y seguridad ecotoxicológica, a escala de laboratorio, semicampo y campo, requeridas para avanzar en el proceso de transferencia, de acuerdo con las directrices de la Organización Mundial de la Salud (OMS) ^38^^,^^39^.

## Ventajas y desventajas de *Leptolegnia chapmanii* como alternativa para el control de *Aedes aegypti*

Como ventajas de *L. chapmanii*, pueden resaltarse la capacidad patógena específica a las larvas de mosquito (previamente comentada) y el prolongado periodo de persistencia (hasta siete semanas) que presenta después de su liberación en recipientes en donde se desarrollan naturalmente las larvas de *A. aegypti*^40^. Otra ventaja que posee *L. chapmanii* es la relativa facilidad con que puede mantenerse en el laboratorio, ya que crece en medios de cultivos simples y de uso rutinario ^41^. Es posible producir biomasa en medio de cultivo a base de semilla de girasol, sustrato económico que ha permitido la reducción de los costos de producción hasta en el 70 % con ventajas adicionales, como una mayor producción de zoosporas ^42^. Este medio de cultivo se prepara licuando 10 g de semillas de girasol en 800 ml de agua destilada. El producto obtenido debe ser filtrado con una gasa doble y esterilizado en autoclave antes de su empleo. La presentación sólida se prepara adicionando 10 g de agar bacteriológico por cada 1.000 ml del volumen final.

El tiempo que demora en generar mortalidad (8-72 horas) puede ser tomado como una desventaja si se compara con el requerido por los larvicidas de síntesis química; sin embargo, es el necesario para infectar (adhesión y penetración por parte de las zoosporas), colonizar y matar a las larvas.

Es importante tener presente que:

- los estados acuáticos de *A. aegypti* se desarrollan en recipientes con capacidad variable, y con aguas de diversa calidad y composición ^(43)^, y
- las zoosporas son las estructuras causantes de la infección de las larvas y su viabilidad se ve afectada por factores ambientales como la temperatura, el pH, la salinidad y el contenido de materia orgánica en el agua, así como por factores externos como la radiación ultavioleta A procedente del sol.

Se ha reportado que estas estructuras toleran temperaturas entre 10 y 35 °C, así como valores de pH entre 4 y 10, y que su viabilidad disminuye con salinidades mayores de 15 ppm o por dosis de radiación ultravioleta A superiores a 19,3 kJ.m-2 ^44^^-^^46^.

Teniendo esto presente, se ha pensado que los productos en desarrollo deben poseer una alta concentración de zoosporas (>108 z/ml), con el fin de asegurar densidades adecuadas en los criaderos de mosquito después de su uso, en busca de tiempos más cortos de respuesta y la mayor eficacia por parte de la herramienta de control.

## Resultados preliminares de un protocolo experimental para el uso de *Leptolegnia chapmanii*

Recientemente se hizo la evaluación de un formulado sólido elaborado en nuestro laboratorio, asumiendo el rol de usuario final. Para esto, se empleó el aislamiento argentino de *L. chapmanii* con número de registro CEP 010, procedente de la colección de hongos entomopatógenos en el Centro de Estudios Parasitológicos y de Vectores (CEPAVE). El mismo fue mantenido en cápsulas de Petri de 90 mm de diámetro, con medio de cultivo PLGa: 1,3 g de peptona de carne, 1,3 g de extracto de levadura, 3 g de glucosa y 10 g de agar bacteriológico por litro de agua destilada. El microorganismo fue mantenido durante siete días a 25 °C y con un fotoperiodo de 12 horas antes de ser utilizado.

**Figura 3:**
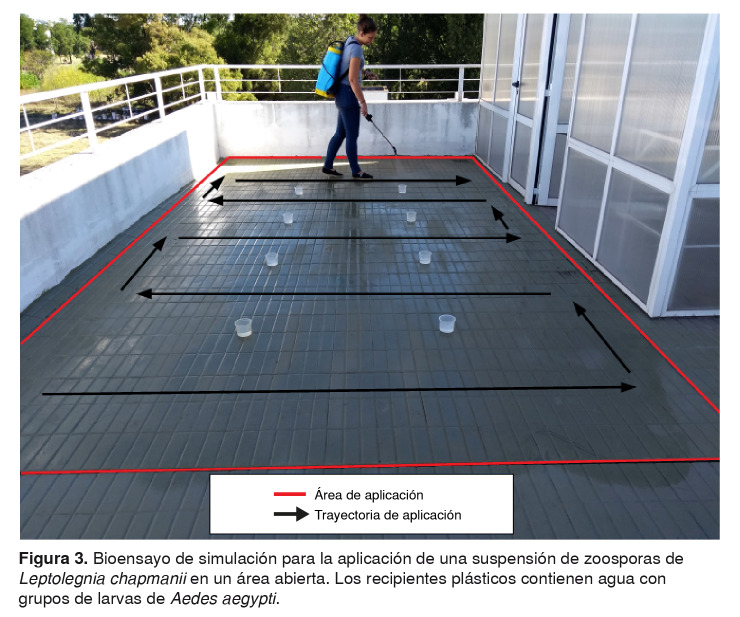
Bioensayo de simulación para la aplicación de una suspensión de zoosporas de *Leptolegnia chapmanii* en un área abierta. Los recipientes plásticos contienen agua con grupos de larvas de *Aedes aegypti*.

1. Preparación del producto para su uso. El medio de cultivo de una cápsula de Petri con *L. chapmanii* fue cortado en seis fragmentos iguales con ayuda de un bisturí, los cuales se introdujeron en un botellón plástico con 8 litros de agua corriente procedente del sistema de abastecimiento de la ciudad. Esta preparación se almacenó durante 48 horas a temperatura ambiente en el interior del Instituto, en espera de obtener zoosporas en suspensión. La concentración de zoosporas se determinó empleando una cámara de Neubauer y un microscopio óptico de contraste de fases.
2. Aplicación de las zoosporas en suspensión. El producto se usó al aire libre, en un área de 15 m² en la terraza del instituto. Para hacerlo, se empleó un pulverizador de espalda de 16 litros que trabaja a presión retenida (ref. 16-M, Giber™, Buenos aires, Argentina) (figura 3). En el área de estudio, se ubicaron ocho recipientes plásticos con capacidad de 200 ml con 100 ml de agua corriente y 10 larvas (L2/L3) de *A. aegypti* procedentes de la colonia de cría establecida en el mismo centro de investigación. Después, los recipientes con las larvas se llevaron al laboratorio y se mantuvieron a 25 °C y con un fotoperiodo de 12 horas.

3. **Resultados**. La presentación sólida de *L. chapmanii* resultó de fácil manejo, y su manipulación fue práctica durante el proceso de preparación y aplicación. Pasadas las 48 horas desde la inmersión del producto en agua, se obtuvieron concentraciones entre 1,3 x 10^3^ y 7 x 10^3^ z/ml; no es clara la razón de las diferencias, aunque corresponden a concentraciones normales según la experiencia que tenemos con este microorganismo.

En el proceso de aplicación, los recipientes recibieron un volumen promedio de 1,4 ml (rango de 0,4 a 2,4 ml), con variaciones por factores ambientales como la dirección y la fuerza del viento durante su uso. Aun cuando la concentración final de zoosporas en los recipientes pudo variar por diferencias obtenidas durante los procesos de preparación y aplicación, la mortalidad se inició antes del primer registro (24 horas después de la aplicación); la mortalidad estuvo entre el 90 y el 100 % después de 72 horas (cuadro 1). En los recipientes que recibieron bajos volúmenes y, por ende, una menor cantidad de zoosporas, el patógeno posiblemente se recicló a partir de aquellas larvas muertas, generando mortalidades altas a las 72 horas después de la aplicación. La mortalidad en los controles negativos varió entre 0 y 20 %.

**Cuadro 1:** Efecto letal de la suspensión de zoosporas de *Leptolegnia chapmanii* sobre *Aedes aegypti* en el bioensayo de simulación de aplicación

	Concentración	Volumen captado por recipiente			Rango de			Porcentaje promedio de mortalidad de larvas			
Réplica	de zoosporas		(ml)		concentración en		Tratamiento				
								
en suspensión				los recipientes									
	Promedio	Mínimo	Máximo				24 horas		48 horas		72 horas		
				(z/ml)	(z/ml)								
														
							Controla	0	-	0	-	0	-	
1	7 x 103				28	- 168	Zoosporasb	100	-	-	-	-	-	
		1,4	0,4	2,4			Control	0	-	10	-	20	-	
2	7 x 103				28	- 168	Zoosporas	51	(41-61)c	81	(70-92)	98	(94-100)	
			
							Control	0	-	0	-	0	-	
3	1,3 x 103				5	- 31	Zoosporas	30	(18-42)	81	(10-92)	98	(94-100)	

a: un recipiente sin el patógeno

b: ocho recipientes tratados

c: intervalo de confianza del 95 %

4. **Conclusiones preliminares.** Esta presentación del producto fue de fácil manipulación por parte del usuario final durante el proceso de preparación, siendo igualmente favorable el uso de la mochila fumigadora para el proceso de aplicación. 

## Limitaciones para la implementación de *Leptolegnia chapmanii* como herramienta en el control de mosquitos

Desde hace décadas, se viene hablando a nivel mundial sobre el ‘manejo integrado de plagas’ y, con el tiempo, se ha hecho evidente la necesidad de vincular estrategias preventivas y culturales con alternativas biológicas y químicas empleadas racionalmente y en respuesta al conocimiento de una situación puntual identificada mediante tareas de vigilancia. Sin embargo, es una realidad que no existe una gran oferta de productos biológicos en el mercado ^47^^,^^48^, y no existe oferta comercial de productos cuya acción se base en relaciones ecológicas, como lo son el parasitismo, la capacidad patógena y la depredación.

Existen grupos de investigación, así como infinidad de publicaciones, que sustentan el potencial de múltiples microorganismos por su acción patógena sobre especies de mosquitos ^49^^,^^50^, pero no existen desarrollos industriales o comerciales con los mismos, lo que sugiere falencias en el proceso de transferencia desde la academia hacia la industria.

Si se analiza el caso puntual de *L. chapmanii*, se encuentra que, durante los más de 40 años de historia desde su descubrimiento, ha sido de interés para grupos puntuales de investigación, siendo acotado su estudio al existir muy pocos aislamientos acordes con la restringida distribución geográfica que se conoce en la actualidad. Es posible que su potencial como herramienta de control se haya visto opacado por la existencia de otros controladores amigables con el ambiente, con mayor historia y grado de desarrollo industrial y comercial. Es el caso de los productos *Bti* basados en las toxinas que produce la bacteria *B. thuringiensis* var. *israelensis* y del oomiceto *Lagenidium giganteum*, el cual llegó a ser producido industrialmente con el nombre comercial de Laginex™, pero que en la actualidad no está disponible en el mercado por confirmarse su afectación sobre mamíferos y otros vertebrados ^51^^-^^53^.

No está clara la razón de la pérdida de interés en *L. chapmanii* por parte de los grupos norteamericanos, pues no hay datos ni publicaciones que permitan controvertir su potencial o que muestren que es perjudicial para los seres vivos o para el medio ambiente.

En cuanto al aislamiento argentino, su estudio durante los últimos 20 años ha presentado una lenta pero coherente evolución, pasando por etapas de análisis básicos en las cuales se determinaron aspectos muy importantes de su biología y ecología, hasta llegar al interés actual por desarrollar productos que puedan ser aplicados en estrategias de control. En el caso brasilero, los aislamientos de *L. chapmanii* son muy recientes (2015) y son material de estudio en la actualidad (Luz CW, comunicación personal, noviembre 15 de 2016).

## Acciones necesarias para lograr una exitosa incorporación de *Leptolegnia chapmanii* a los programas de control de *Aedes aegypti*

Se hace necesario desarrollar procesos de transferencia desde la academia hacia la industria, con el fin de materializar el desarrollo de productos con *L. chapmanii* como ingrediente activo, contando con el soporte operativo, financiero y legal que permita el registro y la comercialización en cumplimiento de las directrices de la OMS y la normatividad existente.

Una acción por desarrollar y que, seguramente, facilitará la implementación de este microorganismo en los programas de control de mosquitos, es la ampliación del conocimiento de la distribución geográfica de la especie, ya que, en países con gobierno federal como Argentina, la legislación es rigurosa en cuanto al uso o la introducción de organismos foráneos en otras provincias. Esta dificultad se presenta igualmente entre naciones; es así que, de confirmarse su presencia natural en diversas áreas geográficas, los desarrollos biológicos basados en *L. chapmanii* podrían emplearse más ampliamente o, en su defecto, los nuevos aislamientos servirían como ingrediente activo para el desarrollo de productos similares y que pudiesen ser implementados de manera local o regional.

Consideramos que la aceptación e inclusión de este tipo de alternativas en los planes de control, dependerán un cien por ciento de los resultados que los productos generen en cuanto a la reducción de las poblaciones de mosquitos. Es por esto que su manipulación y aplicación, deberán llevarse a cabo por personal capacitado, en concordancia con las tareas previas de vigilancia, en las que se dictamine en dónde y cuándo deben hacerse las aplicaciones, esto, con el fin de garantizar un uso racional y los mejores resultados.

## Conclusiones

Existe en la actualidad un soporte científico sólido que permite pensar a *L. chapmanii* como agente biológico con gran potencial para el control de *A. aegypti,* entre otras especies de mosquitos con importancia médica y sanitaria.

El estado actual del desarrollo en torno a la producción de productos formulados basados en el agente entomopatógeno, se encuentra en su etapa inicial; faltan aún estudios de laboratorio, ‘semicampo’ y campo, que permitan determinar la estabilidad, eficacia, eficiencia y seguridad de los productos, así como la viabilidad económica y aceptabilidad en el mercado por parte de los consumidores finales.

El estudio para la determinación de un protocolo de aplicación, generó resultados alentadores. Aun cuando no se conocen perjuicios de esta especie sobre organismos no blanco ni el medio ambiente, es necesario desarrollar, en el futuro, los estudios ecotoxicológicos requeridos por la legislación, siguiendo las directrices de la OMS, con el fin de corroborar su total inocuidad, y avanzar en los procesos de registro y comercialización.

Se hace indispensable ampliar el conocimiento de su distribución geográfica, así como generar procesos de transferencia hacia el sector industrial, con el fin de producir desarrollos masivos, viables económicamente y que puedan ser implementados en los programas de control ejercidos por los entes gubernamentales.
